# Understanding the role of allied health professional support workers with exercise qualifications in the delivery of the NHS Long Term Plan within allied health professional services in England

**DOI:** 10.1136/bmjsem-2023-001625

**Published:** 2023-08-29

**Authors:** Vincent Singh, Katherine Pollard, Rasha Okasheh, John Percival, Fiona Cramp

**Affiliations:** College of Health, Science and Society; School of Health and Social Wellbeing, University of the West of England, Bristol, UK

**Keywords:** Sports & exercise medicine, Exercise rehabilitation, Public health

## Abstract

Demand modelling for the allied health professionals (AHPs) workforce showed that significant expansion would be needed to successfully deliver on the National Health Service (NHS) Long Term Plan. The aim was to explore the use of AHP support workers with exercise qualifications in AHP services and to understand their current and potential role in NHS commissioned AHP services in England. The project had two phases and took place between October 2020 and January 2021. In phase one, an electronic survey was carried out to identify the scope and variation of exercise professionals working in AHP support roles in NHS commissioned services. Semi-structured interviews were conducted in phase two to gain further understanding about the experiences of those involved in AHP commissioned services. Survey data were analysed using descriptive statistics and interview data were qualitatively analysed using thematic analysis. Recorded interviews were transcribed and initially coded. Coding was then refined and themes were identified. Support workers with exercise qualifications made a valued contribution to AHP services and were considered cost-effective in delivering a specialised exercise intervention. AHP support workers contributed to a range of tasks relating to clinical exercise prescription. Collated data highlighted inconsistency in the way AHP support workers with exercise qualifications identified themselves, despite similar roles. Variation existed in the level of autonomy for AHP support workers with exercise qualifications, even within the same NHS Agenda for Change band. Attempts to manage this disparity involved numerous governance processes to ensure safe, high-quality healthcare in the context of delegation to support workers. Limited training and development opportunities and the lack of career progression for support workers were consistently acknowledged as a source of frustration and hindrance to individuals fulfilling their potential. AHP support workers with exercise qualifications have potential to positively impact service delivery providing added value to the NHS workforce.

**WHAT IS ALREADY KNOWN ON THIS TOPIC**

- Disparities and inconsistencies exist in the delivery of exercise as a clinical intervention in the National Health Service in the UK.

**WHAT THIS STUDY ADDS**

- Support workers with exercise qualifications made a valued contribution to allied health professional (AHP) clinical exercise services. Further work is required to develop their professional identity, standardisation of qualifications and career development opportunities.

**HOW THIS STUDY MIGHT AFFECT RESEARCH, PRACTICE OR POLICY**

- The findings may support AHP services to better understand the value that AHP support workers with exercise qualifications/backgrounds offer to clinical exercise services.

## Introduction

There is a growing recognition worldwide that exercise professionals (EPs) with the right training, support and permissions can make a valuable contribution to healthcare.^1–8^ Numerous countries have accredited tertiary qualified allied health professionals (AHPs) working in clinical exercise settings.^9–12^ However, in the UK, EPs are relatively unregulated and levels of qualification and experience needed to serve ‘at-risk’ populations are unclear.^3 8 13^

Furthermore, there is little standardisation of how clinical exercise services are delivered in the National Health Service (NHS) and by whom.^13^ Importantly, there is a lack of recognition in the NHS for appropriately trained clinical exercise professionals despite them being acknowledged as essential for working with people with long-term complex medical conditions.^13^ Distinctive opportunities in the UK, such as specialist exercise instructor courses (as endorsed by the Chartered Institute for the Management of Sport and Physical Activity and the British Association for Cardiovascular Prevention and Rehabilitation^14 15^) and the new status of clinical exercise physiologists, offer excellent potential to develop the workforce in clinical exercise services.^16^

The AHP support worker role has been identified as significant and worthy of greater understanding, prompting AHP managers to consider how AHP support workers are employed and deployed in the NHS.^17^ An AHP support worker in this project is defined as an individual who is working in a non-statutory regulated role under the delegation of a statutory regulated AHP in the NHS. The AHP support workers with a background as an EP (and registered with a recognised exercise and fitness voluntary register), were of specific interest to this work and included exercise scientists, personal fitness trainers, clinical exercise physiologists and rehabilitation therapists working to support AHPs. AHP support workers are often educated to degree level or hold professional qualifications in relevant fields but seldom given the opportunity to demonstrate the full potential of their knowledge and skills.^17^ One potential option to meet the requirements of the NHS Long Term Plan is to develop safe and effective roles for AHP support workers with EP backgrounds.

This project aimed to evaluate the scope and variation in the engagement of AHP support workers with exercise qualifications working in NHS commissioned clinical exercise services to determine the existing provision and potential opportunities that a wider workforce could contribute to patient care and service delivery. Additionally, we aimed to identify the characteristics of the AHP support workforce to inform the key factors to acceptance and integration of AHP support workers in the care pathways.^18^

## Methods

### Study design

A mixed methods evaluation involving two phases was conducted. Both qualitative and quantitative data were gathered in two phases, analysed separately and then both data sets were closely examined to identify converging themes and discrepancies. Synthesising the data contributes to enhanced understanding of the findings and comprehensive results.

### Study advisory panel

Stakeholders were recruited to an advisory panel to review and contribute to the study. The panel members (n=16) included AHPs, academics, service users, representatives from professional bodies, healthcare services, the fitness industry and private companies. The approach was iterative, ensuring continuous update and reflection based on their feedback.

### Survey

In phase one, a survey was co-designed with the panel using the Qualtrics online survey tool (online supplemental appendix 1). The survey’s aim was to identify the characteristics of and determine the different conditions for, and methods of, engaging individuals with exercise qualifications in the care pathways. The survey was piloted locally (n=4) and amendments were made before wider distribution. Both open and closed questions collected data about participants and service characteristics. Exercise referral schemes (ERS) included in this project were defined as a service where an individual is referred by a medical or health professional to a commissioned NHS service that uses physical exercise as a healthcare intervention. The service would offer an assessment of the person’s needs, development of a tailored physical exercise programme, monitoring of progress and follow-up. An option for respondents to confirm whether they were willing to be involved in phase two was included. The survey link was distributed nationally in October 2020 to relevant stakeholders including registered healthcare professionals and AHP support workers with and without exercise qualifications. Following online consent participants could complete the survey. Data were downloaded to secure university servers and analysed using descriptive statistics. Content analysis was used to code open ended questions. Recurring codes were collated and overarching themes reported. A report of the findings was subsequently shared with the advisory panel.

10.1136/bmjsem-2023-001625.supp1 Supplementary data

### Semistructured interviews

In phase two, semistructured interviews were conducted to achieve the following aims:

1. To explore AHPs perception about making referrals to EPs operating as support workers.
2. To explore EPs views about their experience of receiving patient referrals and delivering exercise programmes for service users.

Participants were recruited through purposive sampling method in order to achieve maximum variation, with subsequent invitation to all respondents to participate in the interviews. We wanted to achieve variation based on demographic location, skills mix, roles and responsibilities and career pathways. However, the extent of variation was limited by participant responses. We approached all survey respondents who agreed to be contacted in the qualitative phase of the evaluation as well as individuals identified as potentially rich cases by the advisory panel. In total 11 participants were interviewed.

Topic guides were developed with input from the advisory panel and informed by the survey findings (online supplemental appendix 2). Following a pilot, semistructured individual telephone/Skype interviews averaging 30 min duration were conducted by KP, RO, JP and VS following recorded verbal consent. FC listened to the recording of each interviewer’s first interview and provided feedback to promote consistency of approach. Interviews were transcribed verbatim and thematic analysis was used to identify emergent themes.^19^ Transcripts were initially coded by KP, RO and JP. Coding was then further refined and themes were identified, by JP and RO, a process enhancing analytic rigour.^20 21^ Interviews took place between November 2020 and January 2021.

10.1136/bmjsem-2023-001625.supp2 Supplementary data

## Results

The findings reflect a synthesis of common themes arising from the survey and interviews.

### Survey respondent demographics (n=70)

The majority of respondents were aged between 25 and 44 years old (50%; n=35) and (68.6%; n=48) were women. Overall, 60% of respondents had been qualified for <15 years. Respondents identified themselves by their current role for the purpose of navigating through the survey which did not necessarily correspond with their identification of their primary role. Of the 36 AHP respondents, the majority (64%; n=23) were physiotherapists, one was an occupational therapist and one a podiatrist. Noteworthily, one therapy assistant and three individuals with sports and exercise backgrounds identified themselves as AHPs. Further details of survey respondents roles and years qualified are in table 1.

**Table 1:** Survey respondents’ primary role and years qualified

Current role identified	Primary role	Years qualified (%)	Total
Service manager/lead (n=7, 10%)	AHP or medical professional (n=3)	16–20, n=1 (1.4%)	3 (4.2%)
		26–30, n=1 (1.4%)	
		31–35, n=1 (1.4%)	
	AHP support worker with professional exercise background (n=2)	0–5, n=1 (1.4%)	2 (2.8%)
		26–30, n=1 (1.4%)	
	AHP service lead/manager (n=2)	11–15, n=1 (1.4%)	2 (2.8%)
		>35, n=1 (1.4%)	
Allied health professional (AHP) (n=36, 51.4%)	AHP or medical professional (n=29): Physiotherapist, n=23 Sports/exercise specialist, n=3 Occupational therapist, n=1 Podiatrist, n=1 Therapy assistant, n=1	0–5, n=10 (14.3%)	29 (41.4%)
		6–10, n=4 (5.7%)	
		11–15, n=4 (5.7%)	
		16–20, n=3 (4.2%)	
		21–25, n=2 (2.8%)	
		26–30, n=4 (5.7%)	
		31–35, n=2 (2.8%)	
	AHP support worker (n=2): Physiotherapist, n=1 Sports/exercise specialist, n=1	0–5, n=1 (1.4%)	2 (2.8%)
		11–15, n=1 (1.4%)	
	Exercise professional within NHS commissioned service (n=3): Sports/exercise specialist, n=2 Researcher/practitioner, n=1	0–5, n=1 (1.4%)	3 (4.2%)
		16–20, n=1 (1.4%)	
		21–25, n=1 (1.4%)	
	AHP service lead/manager: physiotherapist	31–35, n=1 (1.4%)	1 (1.4%)
	Local/national commissioner funding exercise referrals: medical professional	16–20, n=1 (1.4%)	1 (1.4%)
**Current role identified**	**Primary role**	**Years qualified (%)**	**Total**
AHP support worker (n=24, 34.3%)	AHP or medical professional (n=2)	16–20, n=1 (1.4%)	2 (2.8%)
		26–30, n=1 (1.4%)	
	AHP support worker with professional exercise background (n=14)	0–5, n=6 (8.6%)	14 (20%)
		6–10, n=2 (2.8%)	
		11–15, n=3 (4.2%)	
		16–20, n=2 (2.8%)	
		26–30, n=1 (1.4%)	
	AHP support worker without professional exercise background (n=5)	0–5, n=1 (1.4%)	5 (7.1%)
		6–10, n=2 (2.8%)	
		11–15, n=2 (2.8%)	
	Exercise professional within NHS commissioned service (n=3)	6–10, n=2 (2.8%)	3 (4.2%)
		11–15, n=1 (1.4%)	

N=67, percentages do not take account of missing data.

NHS, National Health Service.

In total, 11 individuals participated in semi-structured interviews, of which 5 were men. See table 2 for a summary of participant characteristics.

**Table 2:** Interviewee characteristics

I.D.	Age/gender	Role	Setting	Time in post	Qualification(s)	Time since qualifying
AHPSW1	46/M	Exercise rehab instructor	Outpatients: musculoskeletal service	8 months	(a) Level 2 fitness instructor (b) Level 3 personal trainer	(a) 26 years (b) 15 years
AHPSW2	45/M	Exercise instructor	Cardiac and pulmonary rehab team	13 years	(a) Fitness instructor (b) Exercise referral (c) BACPR	(a) 20 years (b) 15 years (c) 10 years
AHPSW3	42/F	Exercise instructor	Outpatients: physiotherapy	2 years 3 months	Level 3 CIMSPA diploma exercise referral	6 years
AHPSW4	45/M	Exercise rehab instructor	Hospitals and a leisure centre	9 months	(a) Sports rehabilitation and injury degree (b) Foundation personal training degree (c) Pilates level 3 (d) Level 3 CIMSPA	11 years
AHPSW5	25/M	Active life trainer	Medium secure mental health hospital (prison transfer patients)	2 years 6 months	(a) BSc (Hons) sports therapy (b) Personal training	4 years
AHP1	49/F	Team lead community physiotherapist	Community-large rural patch	11 months	(a) Undergraduate degree (b) Physiotherapy (c) Masters	(a) 28 years (b) 27 years (c) 19 years
AHP2	57/F	Head of profession for physiotherapy	Mental health partnership trust hospital and community	33 years	Graduate diploma in physiotherapy	37 years
AHP3	53/F	Clinical service manager for musculoskeletal physiotherapy	Musculoskeletal physiotherapy and outpatients: acute and community	2 years	Physiotherapy	25 years
AHP4	25/F	Physiotherapist/rehab instructor lead	Musculoskeletal outpatients department	4 years	Physiotherapy	4 years
AHP5	40/M	Biomechanics specialist podiatrist	Community health centre	17 years	(a) BSc podiatry (b) MSc podiatric biomechanics	17 years
TL1	42/F	Team leader physiotherapist and exercise team	Hospital clinic	18 months	(a) Degree in sports science (b) Level 3 fitness (c) Postgraduate certificate in education	(a) 21 years (b) 11 years (c) 19 years

BACPR, British Association for Cardiovascular Prevention and Rehabilitation; CIMSPA, Chartered Institute for the Management of Sport and Physical Activity.

**Figure 1:**
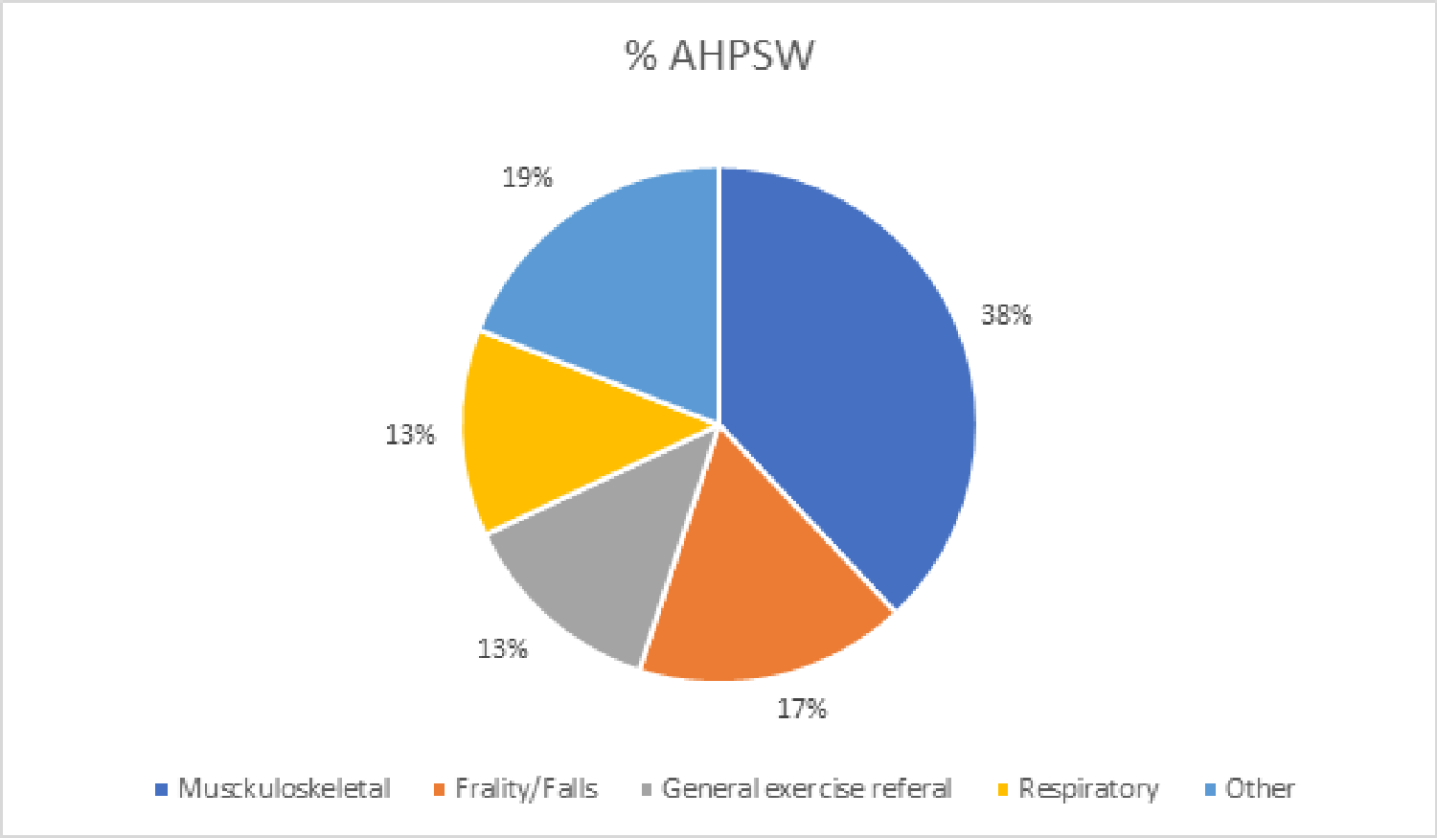
Distribution of allied health professional support workers working in different care pathways. AHPSW, allied health professional support worker.

**Figure 2:**
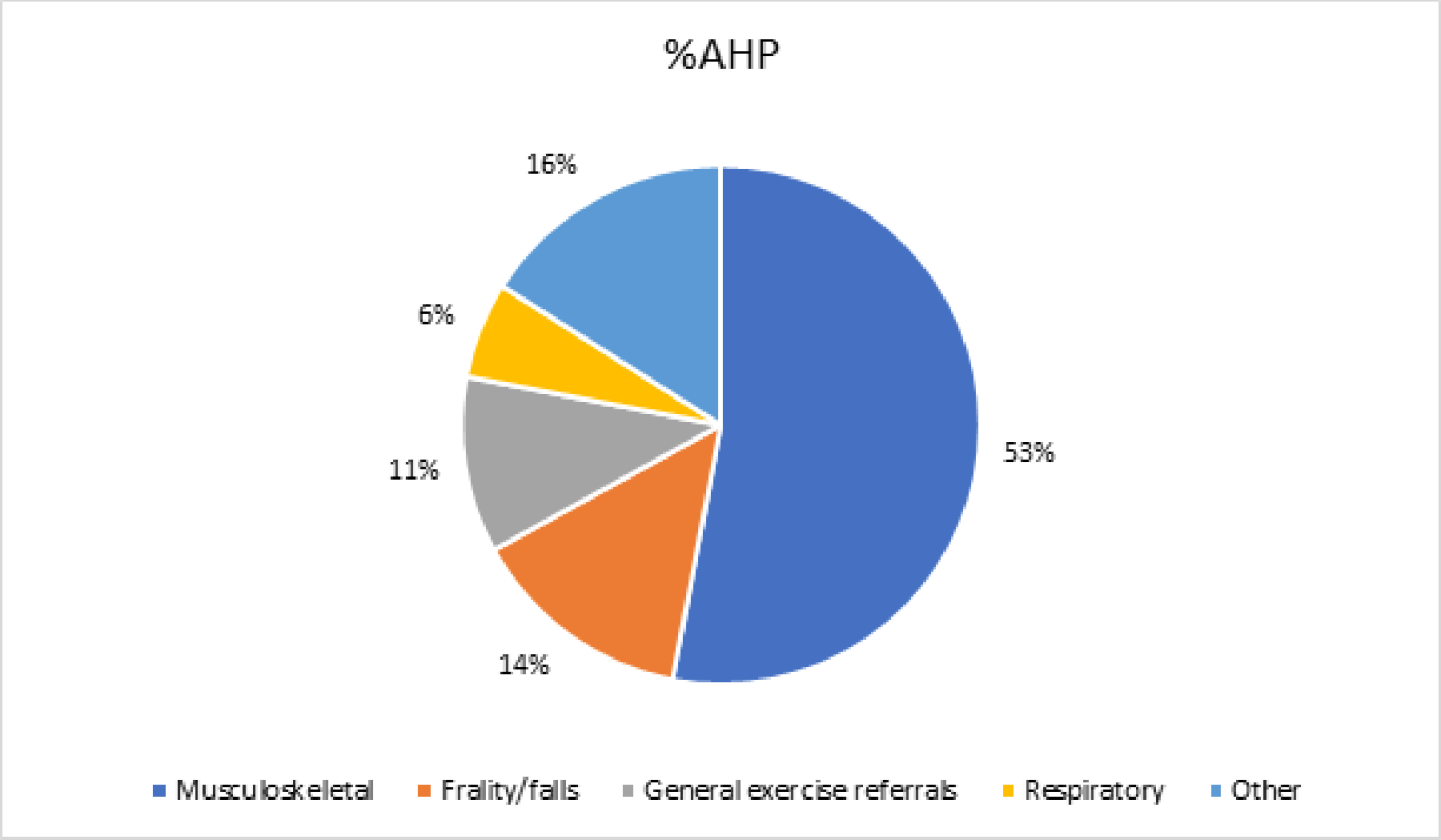
Distribution of allied health professionals (AHPs) working in different care pathways.

The most common care pathway reported within services was musculoskeletal (n=17, 55%); 9 (38%) of the 24 AHP support workers reported practising in this area of care (figure 1), as did 19 (53%) of the 36 AHPs (figure 2). Other pathways included cardiology, frailty/falls, mental health, respiratory, chronic pain, cancer and neurology.

### Service delivery

Most survey respondents (n=57, 81%) worked for the NHS with most services in suburban/urban (n=56, 80%) areas. The AHP support workers with an EP background commonly worked in community leisure facilities, while AHP respondents worked in acute and community settings. This was similar in the qualitative study where a physiotherapist team leader reported that EPs were valued in relation to working alongside community providers to run groups and, in doing so, to provide continuity, “bridge the gap” and “smooth the transition” between hospital and community settings (TL1).

The survey showed an equal split with local authority and acute/primary care trust as the lead agency of ERS. ERS participants were either referred from other services (40%) or recruited opportunistically during consultations (40%). Other processes included patients’ asking to join the scheme and collaboration with the third sector. The initial exercise referral consultations were reportedly booked by an AHP support worker (50%), an AHP (30%) or by patients (20%), with nurses and GPs (general practitioners) also responsible for this. While the survey data suggest a higher proportion of AHP support workers being responsible for the initial exercise referral, the qualitative interviews suggested that the autonomy of the EPS in terms of referrals and triaging has increased over time with support from AHPs:

When I first started… I would work alongside the physiotherapist and so they would start the consultation and make some suggestions, and then from there they would hand the majority of the consultation over to myself… [so I was] kind of weaned away… but we are very supported…. (AHPSW3)

The allocation of work was further described by an interviewee:

It’s the AHPs that would do the triaging to decide if they’re eligible [and] appropriate… then [the case] would be passed to myself or one of my colleagues and we would then do the exercise based assessment … and …plan for the exercise sessions. (AHPSW2)

However, we learnt from interviewees that new work was not always allocated through this triage process. EPs working in hospital settings, for example, would independently and, sometimes, proactively ‘pick up’ cases, in several ways, through their work on wards:

Each ward that we work with have a daily meeting… where you get an opportunity to identify people that you think would be good to work with…The other thing is we have access to notes and all their screenings and assessments, etc. so we can establish an intervention of some sort by looking at notes of people who would benefit from our service, so we can actively seek out people that would benefit… [also] we deliver ward based activities, so Yoga or Tai Chi, [and] you ask people to come and watch, with the view that people will see you and might choose to come and join in. (TL1)

In the analysis of service delivery in the survey, the AHPs (n=26, 72%) reported that the most common working pattern comprised delegation of activities to AHP support workers or following up patients after assessment, triage or referral. There were some examples (n=15, 42%) of collaborative working including the AHP delivering exercise classes with AHP support workers. More than 70% of all the respondents reported AHP support workers delivering (n=57, 81%), monitoring (n=54, 77%) and progressing (n=49, 70%) exercise programmes. In total, 43 (61%) respondents stated that AHP support workers referred patients onwards to exercise or leisure for continuation, while lower numbers indicated their involvement in baseline assessment (n=34, 49%), designing programmes (n=37, 53%) and discharge (n=34, 49%).

In some secondary care settings, AHP support workers worked independently and oversaw the full exercise delivery process:

AHP support workers with exercise qualifications were in charge of the whole of the management of the exercises throughout the class… they have got the ability then to either discharge them, put them on opt in, or have that final decision whether they need to go back to the physiotherapist. (AHP4)

## Governance regarding delegation to AHP support workers with an EP background

The service lead reported that governance for AHP support workers was managed through organisational structures, involving supervision and competency assessment from physiotherapy teams and/or service managers.

They were confident that due governance was in place regarding the delegation of work to AHP support workers. Governance was said to be tied in with a combination of the following tools: the competency framework, required qualifications, clinical supervision, mandatory training, employee appraisal and the employee’s adherence to policies and procedures.

While this appears to be a thin theme, we decided not to integrate it with other themes as it reflects an important aspect of the implantation of AHP support workers within clinical practice. One interpretation of the limited feedback on this aspect is due to limited responses on governance. There were no special governance procedures that consider the uniqueness of the role of AHP support workers so they were embedded within the governance of the organisational structures. This has limited the ability of the organisation to accommodate the flexibility of their roles or to provide appropriate pathways for progression.

### AHP support workers qualifications, competencies and added value

In total, 53 (76%) of survey respondents indicated that at least some of the AHP support workers in their service had some form of exercise-related training or qualification (eg, sport science or sport therapy degree, vocational qualification or assistant practitioner training). The AHP support workers NHS banding ranged from band 2 to 4 (n=20, 83%) with those involved with exercise prescription generally at band 4.

AHP respondents were asked to rate their support workers competencies to manage delegated tasks on a 10-point scale, where a rating of 0 was not competent at all and a rating of 10 was extremely competent. Only 1 of the 36 respondents indicated a level of confidence<5, while 10 (28%) rated their confidence in their AHP support workers’ competencies between 5 and 7 and 25 (69%) between 8 and 10. AHPs rated their perception about support workers’ competence and their confidence in support workers’ ability to carry out their roles between 5 and 7 (n=10, 28%) and between 8 and 10 (n=25, 69%). AHPs (n=16, 44%) stated that support workers freed up the capacity of the statutory regulated staff to engage in leadership and service development.

Several reasons were identified in the interviews for employing AHP support workers with an EP background acknowledging them as being a cost-effective way of providing a specialised intervention to meet a growing demand, while freeing up higher paid AHPs to focus on complex clinical work:

The Trust itself is constantly trying to consider whether we need one expensive member of staff or two or three less expensive members of staff… I prefer to think of that as actually about using people’s skills in the way in which they should be because for me I can have two band 4’s for one Band 7 and if I don't need that leadership level of the Band 7’s in the team I would rather have the workforce on the ground. (AHP1)

A further reason for employing EPs in AHP support workers roles, and one frequently referred to in the context of using staff resources efficiently, was the benefit of ensuring an appropriate skill mix:

There needs to be more of us [AHP support workers with exercise qualifications] … I think a lot of the patients that we see are perfect for us rather than really specialised physios… (AHPSW4)

We depend on our [AHP support workers with exercise qualifications] to do that level of the exercise for us and we [physiotherapists] can then concentrate on assessing those that need it and doing the more complex patients that need physio rehab… (AHP2)

AHP support workers with an EP background are able to spend more time with service users:

more time to spend with the patients… so what we find is [this] patient contact is really, really important in building confidence. (AHP1)

Interviewees highlighted particular abilities, background experience and qualifications as important to be considered in the recruitment of AHP support workers. Specifically, the ability, or potential ability, to work autonomously in carrying out exercise prescription work was identified by a range of interviewees:

We are looking at people who can work autonomously and who are able to do some degree of exercise prescription. (AHP1)

The value of EPs has been particularly demonstrated in mental health services due to their specialised skills in coaching, motivation and engagement. A physiotherapy service leader reported:

One of the biggest things in any form of exercise is engagement and engagement is a massive part of working in mental health as well… engaging people, communication skills, motivation levels… we tended to find that anyone with an exercise background of some sort… can motivate them to do things they don’t want to do. (TL1)

A mixed picture emerged about the necessary qualifications required for AHP support workers with a team leader stating that “the minimum requirement for the [EPs] job is to be level 2 or level 3 fitness qualified”, (TL1) and an AHP indicating “we make sure they [EPs] have at least a Level 3 exercise qualification” (AHP2). The AHP went on to explain that such qualification allowed these AHP support workers to manage the ‘risks’ associated with their vulnerable patients. An AHP support worker believed that being qualified to degree level in sports therapy and registered with a professional society had “allowed [his employer] more confidence” (AHPSW5) in his ability. Specific training varied depending on where the AHP support workers worked, with one AHP indicating that suitably qualified recruits would be trained in the Otago Exercise Programme as this was highly relevant to their ‘frail older’ clients.

### Recognition/lack of recognition (opinion response in survey)

Recognition was reported in terms of aspiration for formal qualification:

AHP support workers without exercise qualification: “I feel I have the capabilities but would like more formal qualifications”.

Lack of recognition was expressed in terms of pay and banding:

AHP support workers without exercise qualification: “I have the capability to provide more targeted patient care, but am in too low a pay band to take on that level of responsibility”.

EP within NHS: “I feel unique in this position compared to my peers and feel strongly that this should be a respected band 5 graduate role in its own right”.

### Underutilisation of AHP support workers’ skills

Underutilised in terms of capacity, scope and skills: The knowledge and skills of AHP support workers with an EP background were sometimes not used optimally. Reasons for this included the predominance of the medical model; lack of recognition of their experience and qualifications; limited understanding and differing perception of their role within the team; and absence of a single representative body for EPs promoting the role.

AHP support workers with exercise qualification: “Currently Instruct 6 classes per week. It is possible to do 3 classes per day”.

AHP support workers with exercise qualification: “I feel with my experience I could deliver exercise programmes to patients. I work in a private setting where I already do this and I feel I’m being held back working for the NHS”.

### Expanding career pathways

Continuing professional development (CPD) opportunities for AHP support workers were reported to comprise in-house training and/or supervision (n=34; 49%) with some respondents indicating external training was also available (n=14; 20%). Half the respondents (n=35; 50%) reported that AHP support workers were able to apply for funding through organisational channels available to all staff. A small proportion reported minimal or no CPD opportunities available for support workers as noted by an AHP support workers with exercise qualification: “I have had very little support to progress in my role, and there is no opportunity for progression”. This was also expressed by an AHP support workers without exercise qualification: “I have found it impossible to achieve funding for training”. Interestingly, 95% of AHPs indicated that there were CPD opportunities for their support workforce compared with 57% of AHP support workers.

## Discussion

This project aimed to gain an understanding about how AHP support workers with exercise qualifications are currently working in NHS commissioned services that include clinical exercise. In these settings, AHP support workers with exercise qualifications are perceived to be cost-efficient and make a valued contribution to NHS service delivery. The collated data highlights inconsistency in the way AHP support workers identify themselves despite similar roles. Variation also exists in the level of autonomy for AHP support workers’ practice even within the same NHS Agenda for Change band. Attempts to manage this disparity involved locally managed governance processes to ensure safe, high-quality healthcare in the context of delegation to AHP support workers. Limited training and development opportunities and the lack of career progression for AHP support workers with a background as an EP was acknowledged as a source of frustration and hindrance to individuals fulfilling their potential.

Our survey and interview findings demonstrate the contribution made by AHP support workers with exercise backgrounds in a wide range of settings where exercise is prescribed. Given the overwhelming evidence supporting the health benefits of physical exercise,^22–27^ it is unsurprising that AHP support workers with exercise backgrounds can effectively contribute to a wide variety of clinical pathways. The broad range of health and well-being benefits available to service users from exercise is mobilised by AHP support workers and thereby offers added value to the NHS workforce.

AHP support workers with EP backgrounds described themselves variously with terms including EP, exercise instructor, rehabilitation instructor or support worker. Similarly, a recent study noted that condition-specific audits in the UK^28 29^ have not attempted to distinguish between clinical exercise staff job titles.^13^ They proposed that this discrepancy is likely due to the level of qualification for delivering clinical exercise services being unclear and suggest the UK considers a formal regulation of clinical exercise physiologists.^13^ Particular merits identified for doing so include raising the education and training level with other AHPs and standardisation for this area of practice. These ambitions would contribute to the NHS achieving standardised, effective and efficient exercise services for long-term health conditions. However, the recognition and resources need to be in place to support such aspirations to enable those AHP support workers to realise their full potential.

Study findings suggest that in addition to exercise prescription qualification, specific training for AHP support workers is needed in specific clinical pathways. For instance, training in the Otago Exercise Programme for those working with frail service users and those at risk of falls, Escape Pain in relation to musculoskeletal conditions and level 4 obesity and diabetes training were specifically identified. The AHP support workers’ practice setting is important to consider when determining the level of qualification required. In ERS and in some trusts in this project, a level 3 qualification was required for working with people with low–moderate risk conditions.^30 31^ This training level was held by only a minority of AHP support workers included in this project which explains why they all worked under the delegation of an AHP. In light of concerns raised in previous research^3^ regarding the competence and effectiveness of those working in higher-risk populations, the clinical exercise physiologist profession^16^ was recently formulated in the UK. This organisation serves as the regulator for this profession and calls on the NHS and healthcare leaders to contribute to professional development and employment of appropriately accredited and regulated clinical exercise physiologists.

Typically, a high proportion of the AHP support workers were responsible for full delivery and progression of exercise programmes for service users. Several AHP support workers also had the authority to refer and discharge service users. There were some roles and responsibilities of AHP support workers that were common although somewhat ad hoc and dependant on the range of activities they carried out in the allied health service in which they were based. The variety of duties that support workers generally perform partly reflects the lack of a common definition of their role as well as the different approaches to workforce design and development models in the NHS trusts.^32^ The NICE (National Institute for Health and Care Excellence) guidelines also recognise this as a gap in the literature and recommends future research to focus on the characteristics of exercise instructors in ERS.^31^ While various duties were carried out by AHP support workers, their roles remained under the delegation of a regulated professional.

The governance processes followed by the trusts participating in this project were managed locally. In addition to having appropriate qualifications, the BHF Exercise Referral Toolkit (2010) recommends that relevant governance arrangements and quality assurance guidelines are followed to ensure a safe and high-quality service. Though there is variation in governance processes across different settings, some practices were common to all trusts, such as supervision and competency assessments. These governance processes and the need for delegation ensure that service users receive safe and effective care which is partly in place because AHP support workers deliver care alongside the regulated, professional workforce but do not hold qualifications that are accredited by a professional regulatory board or regulated by a statutory body.^31^ The advent of the regulation of the UK Clinical Exercise Physiologist by the Professional Standards Authority may serve as a benchmark for those working in clinical exercise services.

Quality of care is an important focus in the NHS, especially with the increasing age of the population and the associated multiple, long-term conditions. It is therefore paramount that the workforce offers safe care with high standards. In this project, AHPs generally expressed confidence in the AHP support workers’ competencies to carry out relevant tasks. As outlined in other multidisciplinary healthcare models, it is important for clinical leaders to build and maintain mutual trust and collaborative relationships with others throughout the healthcare system.^33^ Carter also suggested that appropriate governance processes need to be in place to ensure that all team members can practice ‘at the top of their license’. Given the current lack of professional identity, it is unclear what it means for support workers to be practising ‘at the top of their license’.

Contrary to the safety concerns raised in other areas of support worker practices (eg, nursing^34^), the findings of the current project consistently suggested that AHP support workers make a valued contribution to the lives of service users. In part this may be due to the remarkably low risk of well-designed and appropriately supervised exercise interventions.^3^ Greater confidence in delegated duties to AHP support workers and further research to show how safety could be ensured is needed to support safe and high-quality standards of care.

The study findings indicate that AHP support workers contribute positively to workforce capacity. In line with previous findings, support workers are sometimes recruited to reduce costs through role substitution to free up the regulated healthcare professionals to carry out other duties.^35^ Considering the increasing pressures on the NHS, employing AHP support workers could offer some cost-efficient relief to staffing challenges.

The findings from this project alongside previous studies suggest a need to recognise and support the range of training opportunities available for all AHP support workers and career development pathways. It is argued that the organically developed roles of AHP support workers have contributed to the haphazard preparation for practice and development opportunities.^32^ The recently formulated non-statutory regulated clinical exercise physiologist healthcare science profession in the UK offers career development opportunities for EP occupations.^16^ Unfortunately, the current limited support in the NHS for career development makes it less likely that AHP support workers are prioritised for these training opportunities.

## Limitations

The impact of the COVID-19 pandemic on the NHS and the short recruitment duration, due to the nature of the project funding, are likely to have impacted participation in this project. Particularly, it would be useful to have had input from service users in the interviews and also other AHPs.

## Research implications

This project describes how AHP support workers with exercise qualifications are working in NHS commissioned services. Focus needs to be paid to developing their professional identity, standardising the qualification requirements in general and specific to a range of settings and finally making effective use of resources for CPD opportunities and their career development.

## Data Availability

Data are available upon reasonable request. Data requests are subject to approval of the Chartered Society of Physiotherapy and National Health Service England.
