# Supplementary material for: Understanding the role of allied health professional support workers with exercise qualifications in the delivery of the NHS Long Term Plan within allied health professional services in England
Source: BMJ Open Sport Exerc Med. 2023 Aug 29;9(3):e001625. doi: 10.1136/bmjsem-2023-001625 (PMC10465888; doi:10.1136/bmjsem-2023-001625)
Supplement: Supplementary data [file bmjsem-2023-001625supp001.pdf]

Appendix 1: Survey

Start of Block: Default Question Block

Q1 **Section 1: Participant Information Sheet – On-line Questionnaire** You are invited to take part in an evaluation project taking place at the University of the West of England, Bristol (UWE). It is funded by the Chartered Society of Physiotherapy (CSP). Before you decide whether to take part, it is important for you to understand why the project is being done and what it will involve. Please read the following information carefully and if you have any queries or would like more information please contact Dr Vincent Singh, Faculty of Health and Applied Sciences (HAS), UWE (contact details below).

Who is delivering this project? The project lead is Dr Vincent Singh. Dr Rasha Okasheh, Dr Katherine Pollard and Professor Fiona Cramp are co-Investigators. The team's bios and details of their work are available at https://people.uwe.ac.uk/Person/VincentSingh; https://people.uwe.ac.uk/Person/rashaokasheh; https://people.uwe.ac.uk/Person/katherinepollard; https://people.uwe.ac.uk/Person/fionacramp.

What is the aim of the project? There is a current demand on the health services and therefore a need for workforce transformation in the NHS. It is therefore necessary to develop safe and effective AHP support roles in this area. However, we must first *evaluate how and which support workers are currently involved in services, and how they can contribute to patient care and service delivery*. In particular it is timely to discover where and how support workers are contributing to clinical exercise provision given that this is a core aspect of the work for a number of AHP services; what the existing structures enable support workers' contribution are; and the characteristics of this AHP support workforce are. To help us answer these questions we will be distributing an anonymised on-line questionnaire and also conducting interviews with some participants. The results of our project will be analysed and used in a Report made available on the University of the West of England's open-access repository and will also be

Singh V, *et al. BMJ Open Sp Ex Med* 2024; 9:e001625. doi: 10.1136/bmjsem-2023-001625

made available via Health Education England (HEE) repository. The anonymised results may also be used in conference papers and peer-reviewed academic papers.

Why have I been invited to take part? As you are a health care professional/ support worker/ policymaker or commissioner, we are interested in gaining information about your views. We are therefore inviting you to complete the survey, which will ask you about these opinions and experiences of involving support workers, or being a support worker, in the service.

Do I have to take part? You do not have to take part in this project. It is up to you to decide whether or not you want to be involved. If you do decide to take part, please note that, as the questionnaire is anonymised, it will not be possible for you to withdraw your data once you have completed it.

What will happen to me if I take part and what do I have to do? If you agree to take part, please complete and submit the questionnaire.

What are the benefits of taking part? If you take part in the project, you will be helping us to gain a better understanding of the factors that can influence which support workers are involved in rehabilitation services and how best they can be utilised, both for their own benefit and that of service users.

What are the possible risks of taking part? We do not foresee or anticipate any significant risk to you in completing the survey.

What will happen to your information? All the information that you give will be anonymised when you complete the survey. The survey will be administered using Qualtrics software and will be safely stored on pass-word protected UWE servers. The anonymised data will be stored for an indefinite time period, as they may be used in future evaluation or research projects.

Where will the results of the project be published?

A Report will be written containing our project findings. This Report will be available on the University of the West of England's open-access Research Repository, and HEE. The project funder is CSP and HEE. A hard copy of the Report will be made available to all project participants if you would like to see it. Key findings will also be shared both within and outside

the University of the West of England and HEE. Anonymous and non-identifying direct quotes may be used for publication and presentation purposes.

Who has ethically approved this project? The project has been reviewed and approved by HAS Faculty Research Ethics Committee. Any comments, questions or complaints about the ethical conduct of this project can be addressed to the Research Ethics Committee at the University of the West of England at: Researchethics@uwe.ac.uk

What if something goes wrong? If you have any concerns or complaints about the conduct of this project, please contact Dr Vincent Singh (contact details below).

What if I have more questions or do not understand something? If you would like any further information about the project please contact in the first instance:
Dr Vincent Singh, Senior Lecturer, Department of Allied Health Professionals, HAS, UWE.
Email: Vincent.Singh@uwe.ac.uk Telephone: 01173288897

If you agree to take part in this project, please complete the consent form on the next page.

End of Block: Default Question Block

Start of Block: Block 2

Q2 **Section 2: Consent form**

Q3 I have read the information on the previous page

○ Yes (1)

○ No (2)

Q4 I understand that data I provide will be stored in a secure format, analysed confidentially and that any publication of data will not include personal information.

○ Yes (1)

○ No (2)

Q5 I agree to the University processing my personal data as described above

○ Yes (1)

○ No (2)

Q6 I understand that I can withdraw at any time during the completion of the study, by exiting the web-page (if completing online), and do not need to provide a reason for my withdrawal.

○ Yes (1)

○ No (2)

Q7 I agree to take part in the study

○ Yes (1)

○ No (2)

End of Block: Block 2

Start of Block: Block 3

Q8 **Section 3: Introduction** This survey is part of a larger project which aims to understand the current and potential role of exercise professionals working as AHP support workers in NHS commissioned AHP pathways. The wider project is one of a suite commissioned by Health Education England (HEE) to develop the AHP support workforce and

will contribute to HEE's work to develop and modernised AHP workforce who are enabled at all levels of practice to support the delivery of the NHS Long Term Plan in England.

The survey has been developed to explore the following:

- How AHP support workers with a background as exercise professionals are being deployed in a variety of NHS commissioned AHP managed care pathways.
- The enablers, opportunities and barriers to exercise professionals in AHP support worker roles contributing to services at the height of their capability.
- The enablers, opportunities and barriers to AHP support workers who do not have a background as exercise professionals to be up-skilled in exercise interventions. You are invited to complete this survey if any of the following applies:
- You are a support worker with an exercise background either certified or with vocational training and is working under the delegation of an allied health profession.
- You are an allied health professional working or have experience of working with a support worker with an exercise background either certified or with vocational training and working under the delegation of an allied health profession.
- You are a service manager/leader or commissioner of a service that includes amongst the workforce a support worker with an exercise background either certified or with vocational training and working under the delegation of an allied health profession.

Q9 Please provide your email address below if you would like to be considered to participate in the next phase of this project which will involve the research team contacting you for an interview. The purpose of the interviews will be to gain in-depth information about your experience with the commissioned exercise prescription service.

○ Yes, I consent to be contacted to be considered to participate in an interview (1) ____

○ No, I do not want to be contacted to be considered to participate in an interview (2)

End of Block: Block 3

Start of Block: Block 4

Q10 **Section 4: General questions**

Q11 **What is your age?**

- ☐ Under 18 (1)
- ☐ 18 - 24 (2)
- ☐ 25 - 34 (3)
- ☐ 35 - 44 (4)
- ☐ 45 - 54 (5)
- ☐ 55 - 64 (6)
- ☐ 65 - 74 (7)
- ☐ 75 - 84 (8)
- ☐ 85 or older (9)

Q12 **What is your gender?**

- ○ Male (1)
- ○ Female (2)
- ○ Other (3)
- ○ Rather not say (4)

Q13 **Years of experience in your role since qualification or completed training for role:**

- ○ 0-5 (1)
- ○ 6-10 (2)
- ○ 11-15 (3)
- ○ 16-20 (4)
- ○ 21-25 (5)
- ○ 26-30 (6)
- ○ 31-35 (7)
- ○ Over 35 years (8)

Q14 **Who is your employer?**

- ☐ Local authority (1)
- ☐ NHS (2)
- ☐ NHS provider trust (8)
- ☐ Clinical commissioning group (3)
- ☐ Charity (4)
- ☐ Private (5)
- ☐ Other, please specify (6) ____

Q15 **How would you describe the area in which you work? (you can select more than one option)**

- ▢ Rural (1)
- ▢ Suburban (2)
- ▢ Urban (3)

Q16 **What is your primary role? (you can select more than one option)**

- ▢ Allied health professional or medical health professional (1)
- ▢ AHP support worker with exercise professional background (2)
- ▢ AHP support worker without exercise professional background (3)
- ▢ Exercise professional working in exercise proscription within NHS commissioned service (4)
- ▢ AHP service leader/manger (5)
- ▢ NHS commissioner (6)
- ▢ Local or national commissioner funding exercise referrals (7)

Q17 **How many AHP support workers are there in your immediate service area?**

____

Q18

How many support workers in your immediate service area have a background as an

exercise professional, either qualified or with exercise related vocational training?

____

Q19 **Is exercise experience or qualifications part of the job descriptions for support workers or exercise professionals working in NHS commissioned services where physical exercise is provided and an intervention?**

____

Q20 **Do you think the current number of AHP support workers who are competent to deliver comprehensive exercise interventions is sufficient to meet the demands of the service you are working in/leading/commissioning?**

○ Yes (1)

○ No (2)

○ Don't know (3)

Q21 **What tasks and activities do AHP support workers providing exercise interventions in your service/s undertake?**

- ☐ Baseline assessment (1)
- ☐ Designing programs (2)
- ☐ Delivering programs (3)
- ☐ Monitoring programs (4)
- ☐ Progressing programs (5)
- ☐ Discharging from AHP pathway (6)
- ☐ Referring to exercise/leisure for continuation (7)
- ☐ Other, please specify: (8) ____
- ☐ Don't know (9)

Singh V, *et al. BMJ Open Sp Ex Med* 2024; 9:e001625. doi: 10.1136/bmjsem-2023-001625

Q22 **Where AHP support workers provide exercise interventions, what is the minimum level of exercise qualification required for them to work with this remit in their role?**

○ A relevant degree in sport, exercise or rehabilitation therapy (1)

○ A degree in sport and exercise science (2)

○ A level 3 vocational exercise qualification (3)

○ A level 4 vocational exercise qualification (4)

○ Other vocational exercise training such as Otago (falls), Escape Pain (arthritis), FaME (Stroke) (5)

○ Other, please specify: (6) ____

○ Don't know (7)

Q23 **Do you know what these qualifications entail?**

____

Q24 **In your view, for AHP support workers who do not have a background as an exercise professional what training/development/ qualifications would they need to provide comprehensive exercise interventions?**

____

Q25 **In your service, what are the current arrangements for continuing professional development for AHP support workers?**

____

Q26 **In your service, what approach is adopted for funding continuing professional development for AHP support workers?**

____

Q27 **Do you commission, lead (manage) or work within any exercise referral service?**

- ○ Yes (1)
- ○ No (2)
- ○ Other comments, please specify: (3) ____

Q28 **Is the exercise referral service located within or outside a care pathway?**

- ○ Within (1)
- ○ Outside, please specify where? (2) ____
- ○ Don't know (3)

Singh V, *et al. BMJ Open Sp Ex Med* 2024; 9:e001625. doi: 10.1136/bmjsem-2023-001625

Q29 **In which care pathway is the exercise referral service located? (You can choose more than one)**

- ☐ Musculoskeletal (1)
- ☐ Neurology (2)
- ☐ Cancer (3)
- ☐ Mental Health (4)
- ☐ Cardiology (5)
- ☐ Respiratory (6)
- ☐ Frailty/falls (7)
- ☐ Ante-natal (8)
- ☐ Chronic pain (9)
- ☐ General exercise referral service (10)
- ☐ Disease specific (e.g. stroke, multiple sclerosis, Chronic obstructive pulmonary disease, heart failure), please specify: (11) ____
- ☐ Other, please specify (12) ____

Q30 **Where (setting) is the exercise referral service delivered? (you can choose more than one)**

- ☐ School (1)
- ☐ Clients' workplace (2)
- ☐ Local authority (3)
- ☐ Leisure facility (4)
- ☐ Private facility (5)
- ☐ Home based (6)
- ☐ Outdoor setting, please specify: (7) ________________
- ☐ Community venue (8)
- ☐ Primary care setting (9)
- ☐ Secondary care setting (10)
- ☐ Other, please specify: (11) ________________

Q31 **Who delivers (individuals involved) the exercise referral service?**

____

Singh V, *et al. BMJ Open Sp Ex Med* 2024; 9:e001625. doi: 10.1136/bmjsem-2023-001625

Q32 **Thank you for completing the general questions section, please select the most appropriate option in relation to your primary role to be guided to the appropriate section of the questionnaire.**

- ◯ Support worker, please complete section 5. (1)
- ◯ Allied health professional, please go to section 6 (2)
- ◯ Commissioner, please go to section 7 (3)
- ◯ Service manger/leader, please go to section 8 (4)

End of Block: Block 4

Start of Block: Block 5

Display This Question:

If Thank you for completing the general questions section, please select the most appropriate option... = Support worker, please complete section 5.

Q33 **Section 5: Support workers**

Display This Question:

If Thank you for completing the general questions section, please select the most appropriate option... = Support worker, please complete section 5.

Q34 **How is your post commissioned/ funded?**

____

Display This Question:

If Thank you for completing the general questions section, please select the most appropriate option... = Support worker, please complete section 5.

Q35 **What is your NHS banding?**

- 2 (1)
- 3 (2)
- 4 (3)
- Other, please specify: (4) ____
- Don't know (5)

Display This Question:

If Thank you for completing the general questions section, please select the most appropriate option... = Support worker, please complete section 5.

Singh V, *et al. BMJ Open Sp Ex Med* 2024; 9:e001625. doi: 10.1136/bmjsem-2023-001625

Q36 In which care pathway are you currently practicing? (You can choose more than one)

- Musculoskeletal (1)
- Neurology (2)
- Cancer (3)
- Mental Health (4)
- Cardiology (5)
- Respiratory (6)
- Frailty/falls (7)
- Ante-natal (8)
- Chronic pain (9)
- General exercise referral service (10)
- Disease specific (e.g. stroke, multiple sclerosis, Chronic obstructive pulmonary disease, heart failure), please specify: (11) ____
- Other, please specify (12) ____

Display This Question:

If Thank you for completing the general questions section, please select the most appropriate option... = Support worker, please complete section 5.

Q37 **What type of clients do you work with and which of these do you support with exercise interventions?**

____

Display This Question:

If Thank you for completing the general questions section, please select the most appropriate option... = Support worker, please complete section 5.

Q38 **In what settings are you currently practicing? (you can choose more than one)**

- ▢ Community service e.g. GP surgery, community clinic (1)
- ▢ Acute hospital in-patient (2)
- ▢ Acute hospital out-patient (3)
- ▢ Local authority leisure facility (6)
- ▢ Home-based (7)
- ▢ Sports club (8)
- ▢ Private leisure facility (9)
- ▢ Community venue, e.g. church hall (10)
- ▢ Outdoor settings/ Green exercise, e.g. green gyms (11)
- ▢ Click to write Choice 12 (14)

Display This Question:

If Thank you for completing the general questions section, please select the most appropriate option... = Support worker, please complete section 5.

Q39 What is your current level of qualification/training in exercise prescription?

- ☐ A relevant degree in sport, exercise or rehabilitation therapy (1)
- ☐ A relevant degree in sport and exercise science (2)
- ☐ level 3 vocational exercise qualification (3)
- ☐ level 4 vocational exercise qualification (4)
- ☐ Other vocational exercise training such as Otago (falls), Escape Pain (arthritis), FaME (Stroke) (5)
- ☐ Other, please specify: (6) ____
- ☐ Don't know (7)

Display This Question:

If Thank you for completing the general questions section, please select the most appropriate option... = Support worker, please complete section 5.

Q40 **Do you know what these qualifications entail?**

____

Display This Question:

If Thank you for completing the general questions section, please select the most appropriate option... = Support worker, please complete section 5.

Q41 **Does your role involve exercise prescription i.e. delegated authority to design, prescribe, and deliver an exercise programme?**

- Yes (1)
- No (2)
- Other comments, please specify: (3) ____

Display This Question:

If Thank you for completing the general questions section, please select the most appropriate option... = Support worker, please complete section 5.

Q42 **Does your role involve delivery of a prescribed exercise programme i.e. you support someone with an exercise programme that has been designed/prescribed by someone else and delegated to you to implement?**

- Yes (1)
- No (2)
- Other comments, please specify: (3) ____

Display This Question:

If Thank you for completing the general questions section, please select the most appropriate option... = Support worker, please complete section 5.

Singh V, *et al. BMJ Open Sp Ex Med* 2024; 9:e001625. doi: 10.1136/bmjsem-2023-001625

Q43 **Do you have delegated authority to alter and progress exercise programme i.e. you do not have to discuss or seek authority from a registered healthcare professional to do this?**

○ Yes (1)

○ No (2)

○ other comments. please specify: (3) ____

Display This Question:

If Thank you for completing the general questions section, please select the most appropriate option... = Support worker, please complete section 5.

Q44 **What special competencies related to exercise interventions, that enhances the patient management and improve their experience do you think you have?**

____

Display This Question:

If Thank you for completing the general questions section, please select the most appropriate option... = Support worker, please complete section 5.

Q45 **Do you work within an exercise referral scheme (a formal program whereby a primary health care professional refers a patient to a fitness program, often based within the community)?**

○ Yes (1)

○ No (2)

Singh V, *et al. BMJ Open Sp Ex Med* 2024; 9:e001625. doi: 10.1136/bmjsem-2023-001625

Display This Question:

If Do you work within an exercise referral scheme (a formal program whereby a primary health care pr... = Yes

And Thank you for completing the general questions section, please select the most appropriate option... = Support worker, please complete section 5.

Q46 **Who is the lead agency for the scheme?**

- local authority (1)
- Voluntary sector (2)
- Primary care trust (3)
- Acute trust (4)
- University (5)
- Private sector (6)
- Joint local authority and primary care trust (7)
- Other, please specify: (8) ____

Display This Question:

If Do you work within an exercise referral scheme (a formal program whereby a primary health care pr... = Yes

And Thank you for completing the general questions section, please select the most appropriate option... = Support worker, please complete section 5.

Q47 **How are participants recruited to the scheme?**

- ◯ Opportunistically in a consultation (1)
- ◯ New patient consultation (2)
- ◯ Health screening via existing condition clinic e.g. asthma (3)
- ◯ Existing disease register e.g. chronic heart disease (4)
- ◯ Via advertising e.g. in practice (5)
- ◯ Patient initiated request (6)
- ◯ Other, please specify: (7) ____

Display This Question:

If Do you work within an exercise referral scheme (a formal program whereby a primary health care pr... = Yes

And Thank you for completing the general questions section, please select the most appropriate option... = Support worker, please complete section 5.

Q48 **Who is responsible for booking the initial exercise referral consultation?**

- ◯ GP (1)
- ◯ Allied health professional (2)
- ◯ Nurse (3)
- ◯ Patient (4)
- ◯ Exercise professional (5)
- ◯ Practice receptionist (6)
- ◯ Other, please specify: (7) ____

Singh V, *et al. BMJ Open Sp Ex Med* 2024; 9:e001625. doi: 10.1136/bmjsem-2023-001625

Display This Question:

If Thank you for completing the general questions section, please select the most appropriate option... = Support worker, please complete section 5.

Q49 **What type of support do you receive from your clinical team?**

- ○ Mentoring (1)
- ○ Supervision and feedback (2)
- ○ In house training (3)
- ○ Other, please specify: (4) ____

Display This Question:

If Thank you for completing the general questions section, please select the most appropriate option... = Support worker, please complete section 5.

Q50 **Do you believe that level of supervision and feedback you receive is appropriate to the exercise interventions being delegated to you?**

- ○ Yes, it is consistently appropriate (1)
- ○ Not always appropriate (2)
- ○ I do not think I receive sufficient supervision and feedback (3)
- ○ I do not receive any supervision or feedback (4)

Display This Question:

If Thank you for completing the general questions section, please select the most appropriate option... = Support worker, please complete section 5.

Q51 **Have you received specific training in all or any of the following domains in order to work in your current role? Choose all that apply.**

- ◯ Specialized clinical training related to the clinical population you work with (e.g stroke, COPD, chronic pain etc.) (1)
- ◯ Patient motivation and engagement (2)
- ◯ health behaviour change techniques (3)
- ◯ Communication (4)
- ◯ Goal setting (5)
- ◯ Clinical skill (6)
- ◯ Other, please specify: (7) ____
- ◯ No specific training received (8)

Display This Question:

If Thank you for completing the general questions section, please select the most appropriate option... = Support worker, please complete section 5.

Q52 **How do you feel about the current scope of practice in your role?**

- ◯ I have the capability and qualifications to deliver more treatment support to patients than I currently provide (1)
- ◯ I am working at about the right level for my existing capability and qualifications (2)
- ◯ I am working beyond my capability and level of qualifications (3)

Display This Question:

If Thank you for completing the general questions section, please select the most appropriate option... = Support worker, please complete section 5.

Q53 **Please state why you feel this to be the case?**

____

Singh V, *et al. BMJ Open Sp Ex Med* 2024; 9:e001625. doi: 10.1136/bmjsem-2023-001625

End of Block: Block 5

Start of Block: Block 6

Display This Question:

If Thank you for completing the general questions section, please select the most appropriate option... = Allied health professional, please go to section 6

Q54 **Section 6: Allied Health professionals**

Display This Question:

If Thank you for completing the general questions section, please select the most appropriate option... = Allied health professional, please go to section 6

Q55 **What is your professional role?**

____

Display This Question:

If Thank you for completing the general questions section, please select the most appropriate option... = Allied health professional, please go to section 6

Q56 **Are you currently involved in exercise prescription or delivery for patients?**

◯ Yes (1)

◯ No (2)

Display This Question:

If Thank you for completing the general questions section, please select the most appropriate option... = Allied health professional, please go to section 6

Q57 **In which care pathway are you currently practicing? (You can choose more than one)**

- ▢ Musculoskeletal (1)
- ▢ Neurology (2)
- ▢ Cancer (3)
- ▢ Mental Health (4)
- ▢ Cardiology (5)
- ▢ Respiratory (6)
- ▢ Frailty/falls (7)
- ▢ Ante-natal (8)
- ▢ Chronic pain (9)
- ▢ General exercise referral service (10)
- ▢ Disease specific (e.g. stroke, multiple sclerosis, Chronic obstructive pulmonary disease, heart failure), please specify: (11) ____
- ▢ Other, please specify (12) ____

Display This Question:

If Thank you for completing the general questions section, please select the most appropriate option... = Allied health professional, please go to section 6

Q58 **What type of patients/ clinical populations do you work with?**

____

Display This Question:

If Thank you for completing the general questions section, please select the most appropriate option... = Allied health professional, please go to section 6

Q59 **In what settings are you currently practicing? (you can choose more than one)**

- ▢ Primary care (1)
- ▢ Acute hospital (in-patient) (2)
- ▢ Acute hospital (out-patient) (3)
- ▢ Local authority leisure facility (6)
- ▢ Home based (7)
- ▢ Sports club (8)
- ▢ Private leisure facility (9)
- ▢ Community venue, e,g, church hall (10)
- ▢ Outdoor settings/ green exercise, e.g. green gyms (11)
- ▢ Other, please specify: (12) ____

Display This Question:

If Thank you for completing the general questions section, please select the most appropriate option... = Allied health professional, please go to section 6

Q60 **Do you work within an exercise referral scheme?**

◯ Yes (1)

◯ No (2)

Display This Question:

If Thank you for completing the general questions section, please select the most appropriate option... = Allied health professional, please go to section 6

And Do you work within an exercise referral scheme? = Yes

Q61 **Who is the lead agency for the scheme?**

◯ Local authority (1)

◯ Voluntary sector (2)

◯ Primary care trust (3)

◯ Acute trust (4)

◯ Community provider (eg: Sirona, Virgin care) (9)

◯ University (5)

◯ Private sector (6)

◯ Joint local authority and PCT (7)

◯ Other, please specify: (8) ____

Display This Question:

If Thank you for completing the general questions section, please select the most appropriate option... = Allied health professional, please go to section 6

And Do you work within an exercise referral scheme? = Yes

Singh V, *et al. BMJ Open Sp Ex Med* 2024; 9:e001625. doi: 10.1136/bmjsem-2023-001625

Q62 **How are participants recruited to the scheme?**

- ◯ Opportunistically in a consultation (1)
- ◯ New patient consultation (2)
- ◯ Health screening via existing condition clinic e.g. asthma (3)
- ◯ Via existing disease registers e.g. Chronic heart disease (4)
- ◯ Via advertising e.g. in practice patient initiated request (5)
- ◯ Other,please specify: (6) ____

Display This Question:

If Thank you for completing the general questions section, please select the most appropriate option... = Allied health professional, please go to section 6

And Do you work within an exercise referral scheme? = Yes

Q63 **Who is responsible for booking the initial exercise referral consultation?**

- ◯ GP (1)
- ◯ Allied health professional (2)
- ◯ Nurse (3)
- ◯ Patient (4)
- ◯ Exercise professional (5)
- ◯ Practice receptionist (6)
- ◯ Other, please specify: (7) ____

Display This Question:

If Thank you for completing the general questions section, please select the most appropriate option... = Allied health professional, please go to section 6

Singh V, *et al. BMJ Open Sp Ex Med* 2024; 9:e001625. doi: 10.1136/bmjsem-2023-001625

Q64 **Do you work with AHP support workers?**

○ Yes (1)

○ No (2)

Display This Question:

If Thank you for completing the general questions section, please select the most appropriate option... = Allied health professional, please go to section 6

Q65 **Could you describe the pattern of working with AHP support workers, e.g. Co working on cases, Delegation.**

____

Display This Question:

If Thank you for completing the general questions section, please select the most appropriate option... = Allied health professional, please go to section 6

Q66 **What level of training/ education do the AHP support workers you delegate exercise interventions to have?**

○ A relevant degree in sport, exercise or rehabilitation therapy (1)

○ A degree in sport and exercise science (2)

○ A level 3 vocational exercise qualification (3)

○ A level 4 vocational exercise qualification (4)

○ Other vocational exercise training such as Otago (falls), Escape Pain (arthritis), FaME (Stroke) (5)

○ Other, please specify: (6)

○ Don't know (7)

Display This Question:

If Thank you for completing the general questions section, please select the most appropriate option... = Allied health professional, please go to section 6

Q67 **What percentage of the support workers you work with do you consider to be exercise professionals?**

____

Display This Question:

If Thank you for completing the general questions section, please select the most appropriate option... = Allied health professional, please go to section 6

Q68 **Do your support workers with a background as an exercise professional have ddelegated authority to design and prescribe an exercise programme?**

○ Yes (1)

○ No (2)

Display This Question:

If Thank you for completing the general questions section, please select the most appropriate option... = Allied health professional, please go to section 6

Q69 **Do your support workers with a background as an exercise professional have delegated authority to deliver an exercise programme?**

○ Yes (1)

○ No (2)

Singh V, *et al. BMJ Open Sp Ex Med* 2024; 9:e001625. doi: 10.1136/bmjsem-2023-001625

Display This Question:

If Thank you for completing the general questions section, please select the most appropriate option... = Allied health professional, please go to section 6

Q70 **Do your support workers with a background as an exercise professional have delegated authority to alter and progress an exercise programme?**

○ Yes (1)

○ No (2)

Display This Question:

If Thank you for completing the general questions section, please select the most appropriate option... = Allied health professional, please go to section 6

Q71 **Do your support workers without a background as an exercise professional have delegated authority to design and prescribe an exercise programme?**

○ Yes (1)

○ No (2)

Display This Question:

If Thank you for completing the general questions section, please select the most appropriate option... = Allied health professional, please go to section 6

Q72 **Do your support workers without a background as an exercise professional have delegated authority to deliver an exercise programme?**

○ Yes (1)

○ No (2)

Display This Question:

If Thank you for completing the general questions section, please select the most appropriate option... = Allied health professional, please go to section 6

Singh V, *et al. BMJ Open Sp Ex Med* 2024; 9:e001625. doi: 10.1136/bmjsem-2023-001625

Q73 **Do your support workers without a background as an exercise professional have delegated authority to alter and progress an exercise programme?**

- Yes (1)
- No (2)

Display This Question:

If Thank you for completing the general questions section, please select the most appropriate option... = Allied health professional, please go to section 6

Q74 **What impact does the involvement of AHP support workers with exercise professional background have on your clinical practice? (select any or all that apply)**

- Frees up capacity in the statutory registered workforce to work at higher levels (1)
- Frees up the statutory registered workforce to spend greater time with complex patients (2)
- Frees up the statutory registered workforce to engage more in leadership and service development. (3)
- Other, please specify: (4) ____

Display This Question:

If Thank you for completing the general questions section, please select the most appropriate option... = Allied health professional, please go to section 6

Q75 **In general how confident do you feel in the competencies of AHP support workers with an exercise professional background in managing the tasks delegated to them?**

- 0 (0)
- 1 (1)
- 2 (2)
- 3 (3)
- 4 (4)
- 5 (5)
- 6 (6)
- 7 (7)
- 8 (8)
- 9 (9)
- 10 (10)

Display This Question:

If Thank you for completing the general questions section, please select the most appropriate option... = Allied health professional, please go to section 6

Q76 **In general how would you describe the competency of AHP support workers with an exercise professional background in the performance of the activities delegated to them?**

- ◯ 0 (0)
- ◯ 1 (1)
- ◯ 2 (2)
- ◯ 3 (3)
- ◯ 4 (4)
- ◯ 5 (5)
- ◯ 6 (6)
- ◯ 7 (7)
- ◯ 8 (8)
- ◯ 9 (9)
- ◯ 10 (10)

End of Block: Block 6

Start of Block: Block 7

Display This Question:

If Thank you for completing the general questions section, please select the most appropriate option... = Commissioner, please go to section 7

Q77 **Section 7: Commissioners**

The Allied Health support workers are a group of unregulated support workers who help Allied Health Professionals deliver the services they are responsible for.

Display This Question:

If Thank you for completing the general questions section, please select the most appropriate option... = Commissioner, please go to section 7

Q78 What type of data do you collect to show how AHP support workers are deployed?

____

Display This Question:

If Thank you for completing the general questions section, please select the most appropriate option... = Commissioner, please go to section 7

Q79 What impact do you think AHP support workers with an exercise professional background have on the management of ppatients particularly in relation to fulfilling the objectives of the NHS long term plan?

____

Display This Question:

If Thank you for completing the general questions section, please select the most appropriate option... = Commissioner, please go to section 7

Q80 Is funding different for AHP support workers delivering exercise programmes compared to those who are not working in the deliver of exercise programmes?

____

End of Block: Block 7

Start of Block: Block 8

Display This Question:

If Thank you for completing the general questions section, please select the most appropriate option... = Service manger/leader, please go to section 8

Q81 **Section 8: AHP service mangers**

Display This Question:

If Thank you for completing the general questions section, please select the most appropriate option... = Service manger/leader, please go to section 8

Q82 **What is the proportion of non-registered workforce to the registered work force in your service area?**

○ Less than 25% (1)

○ 25%-35% (2)

○ above 35% (3)

Display This Question:

If Thank you for completing the general questions section, please select the most appropriate option... = Service manger/leader, please go to section 8

Q83 **What is the range of NHS banding of your AHP support workers who are involved in providing exercise prescription in your service?**

____

Display This Question:

If Thank you for completing the general questions section, please select the most appropriate option... = Service manger/leader, please go to section 8

Q84 **How are AHP support workers' activities remunerated?**

○ Block contracts (1)

○ Cost per case (2)

○ Other, please specify: (3) ____

Display This Question:

If Thank you for completing the general questions section, please select the most appropriate option... = Service manger/leader, please go to section 8

Q85 **What are the requirements in terms of qualification for personnel involved in the delivery of exercise prescription?**

____

____

____

____

____

Display This Question:

If Thank you for completing the general questions section, please select the most appropriate option... = Service manger/leader, please go to section 8

Q86 **What are the governance arrangements for support workers in your services?**

____

____

____

____

____

Display This Question:

If Thank you for completing the general questions section, please select the most appropriate option... = Service manger/leader, please go to section 8

Q87 **What are the continuing professional development activities that are available to support workers working in exercise prescription, both within and external to your service, that is supported by the service?**

____

____

____

____

Display This Question:

If Thank you for completing the general questions section, please select the most appropriate option... = Service manger/leader, please go to section 8

Q88 **What are the career pathways available for support workers working in exercise prescription?**

Display This Question:

If Thank you for completing the general questions section, please select the most appropriate option... = Service manger/leader, please go to section 8

Q89 **Do you have established targets for increasing the support worker workforce in line with the increase in the registered workforce?**

- Yes, could you please describe them: (1) ________________
- No, what might be the barriers? (2) ________________
- Under development, could you briefly describe the plan: (3) ________________

End of Block: Block 8

Singh V, *et al. BMJ Open Sp Ex Med* 2024; 9:e001625. doi: 10.1136/bmjsem-2023-001625
