# Supplementary material for: Understanding the role of allied health professional support workers with exercise qualifications in the delivery of the NHS Long Term Plan within allied health professional services in England
Source: BMJ Open Sport Exerc Med. 2023 Aug 29;9(3):e001625. doi: 10.1136/bmjsem-2023-001625 (PMC10465888; doi:10.1136/bmjsem-2023-001625)
Supplement: Supplementary data [file bmjsem-2023-001625supp002.pdf]

Appendix 2: Interview topic guide

Interviews with AHPs, sports and exercise professionals and people who use the service

Steps to follow:

1. Team member to contact participant to check that they are still willing to participate.
2. If yes, send them PIS and Consent forms to read and arrange suitable time for interview.
3. Arrange a time to phone interviewee and then do so using skype for business to make the call
4. Commence call, check participant has read information sheet and answer any questions they may have. Confirm that the participant agrees to the interview being recorded.
5. Start the recording.
6. Ensure consent form is signed prior to start of interview. Remind participant of key parts of the information sheet: that interview will be recorded, that they can stop the interview at any time/skip any questions they wish to, everything they say is confidential, any extracts reported from the interview will be anonymous.
7. Confirm basic biographic details obtained from survey: Age, gender, healthcare practitioner role, qualifications, employment setting, duration of current employment, years since qualification.
8. Check that the interviewee understands how we are defining AHP support workers/exercise professionals (see appendix A)
9. Interview: follow interview topic guide (table 1) for the appropriate role of the participant.
10. After the interview has ended, stop recoding and then upload the saved recording to the OneDrive folder (CSP project)

Aims

The next phase of the project aims to gain further understanding about the experiences of those involved in AHP commissioned services.

1. The research team will conduct interviews with AHPs to explore their perceptions about any relevant issues when delegating exercise interventions to sports and exercise professionals who are operating as support workers.
2. Interviews with sport and exercise professionals will provide further insights into their views about their experience of working under the delegation of a registered AHP and delivering exercise programmes for service users.
3. The service user's opinions about their experiences with sport and exercise professionals working as support workers will also be captured during interviews.

Participant	Sample questions and prompts
AHP service lead/service manager/team lead	What is your role in the service?<br><br>How do you make decisions on who to employ in a service that has a focus on exercise interventions as a core part of service delivery?

	How is the budget apportioned to the workforce who provide the exercise component of prevention/rehabilitation interventions?<br><br>Can you tell me how sport and exercise professionals were initially brought in to your service as AHP support workers? What were the key drivers for this?<br><br>For your support workers who have a background as exercise professionals how was the banding into which they are employed determined?<br><br>Where there is an explicit focus on exercise as an intervention, how effective do you think your service is, both for your trust and the service user?<br>Note: try to distinguish between clinical exercise and rehab exercise.<br><br>What governance processes do you have in place to ensure that the exercise interventions delegated to your support workers are safe and effective?<br><br>Do you think your support workers with a background as an exercise professional could offer more in your service? Why is that?<br><br>How do you know whether your support workers who are delivering exercise interventions are having an impact on the service? Do you have any explicit data that demonstrates this?<br><br>Is there scope for CPD or career progression for support workers?<br><br>Is there anything else you would like to tell us about the role and conditions for AHP support workers involved in exercise intervention in your organisation?
AHP	What is your role in your service?<br><br>How confident are you in your knowledge and practical application of exercise prescription for clinical populations?<br><br>How are clinical exercise interventions delivered and prioritised in your work setting? How are decisions made about who delivers exercise interventions? Do you have a triage system and if so, what are the inclusion / exclusion criteria and how are they matched with the right patients?<br><br>Are there any education / training opportunities in clinical exercise delivery? If yes, what are these and who has access to these opportunities?

	Can you tell me how sport and exercise professionals were initially brought in to your service as AHP support workers? What were the key drivers for this?<br><br>Are you aware of any barriers to delegating to AHP support workers to provide tailored exercise interventions?<br>What do you think the effect on the service is where AHP support workers undertake delegated exercise interventions? Is there any evidence to support your views?<br><br>Do you have anything else you would like to say about the role of clinical exercise in your service or sport and exercise professionals working as AHP support workers to specifically deliver this?
AHP support worker<br>Note: please check if they self- identify as an exercise professional but are working as a support worker	Which of the following titles do you feel best describes your role; exercise professional or support worker or would you like to offer another description? Why do you see yourself as that?<br><br>Do you have a background as an exercise professional?<br>If yes, how does this background contribute to your work as an AHP support worker? Probe: how long they have worked in the role.<br><br>Do you have a vocational qualification in sport and exercise or other relevant qualification? If yes, how does this background contribute to your work as an AHP support worker<br><br>How confident are you in your knowledge and practical application of exercise prescription for clinical populations? Why is that?<br><br>How have you developed your skills to enable you to become an AHP support worker? How was that supported? What other training would you like to be able to access?<br><br>If you do not have an exercise professional background, how have you acquired these skills?<br><br>How are clinical exercise interventions delivered and prioritised in your work setting? How are decisions made about who delivers the exercise intervention?<br><br>Can you tell me a bit about the patient groups that you encounter clinically and your experience in the delivery of clinical exercise for these patients?<br><br>Do you aspire to develop your skills further? (*Probe for details)*.

	Do you feel that the role you are in enables you to work at the top of your capability to provide exercise interventions?<br>Why is that?<br><br>What more do you feel you could bring to your service in terms of your personal scope and the qualifications you hold? What prevents you from doing that?<br><br>Do you aspire to become a registered AHP?<br>If so, what would enable you to do that? What might the barriers be?<br><br>Do you have anything else you would like to say about the provision of clinical exercise interventions in the service you work in<br><br>Prompts:<br>If exercise professionals indicate they could do more worth really exploring what limits this
Service user	What was the reason for the consultation with AHP support worker?<br><br>Who/how was the referral made?<br><br>Can you tell me a bit about the course of exercise prescription that you received? Prompts: number of appointments, duration of individual appointments and overall course of treatment and type of support they received.<br><br>Did you find the treatment helpful? If yes, how and if no what could have been better? Probe: find out in which setting this was eg: hospital.<br><br>Did the AHP support worker spend time with you to understand your capabilities which may be due to the condition or treatment?<br><br>Were you involved in any of the decision making relating to exercise service?<br><br>Would you recommend the service to others? Why?<br><br>Is there anything else you would like to tell us about your experience of treatment with the support worker?<br><br>Prompts: Were you confident that the AHP support worker knew enough about your condition? Were you satisfied with the care that you received?

Table 1: Interview topic guide

Appendix A: Definition of Terms

Physical activity, sport and exercise in the context of health is broad ranging. The following are definitions of the terms used in this project specific to the overall aims and objectives.

1. Allied Health Professional (AHP) support worker: individual who is working in a non-statutory regulated role under the delegation of a statutory regulated AHP.

2. AHP commissioned service: planning, agreeing and monitoring of populations health-needs assessment through to the clinical based design of patient pathways where exercise prescription is used.

3. Care pathway: a way of setting out a process of best practice to be followed in the treatment of a patient or client with a particular condition or with particular needs. It is a distillation of the best available expert opinion on the care process and should be evidence based.

4. Exercise referral scheme: where an individual is referred by a medical or health professional to a commissioned NHS service that uses physical exercise as a healthcare intervention. The service would offer an assessment of the person’s needs, development of a tailored physical exercise programme, monitoring of progress and a follow-up.

5. Exercise professional: an individual who is registered with a recognised exercise and fitness voluntary register such as the Register of Exercise Professionals, Chartered Institute for the Management of Sport and Physical Activity. The exercise professionals we are interested in interviewing may include exercise scientists, personal fitness trainers, clinical exercise physiologists and rehabilitation therapists.
